# *C. elegans* spermatozoa lacking *spe-45* are incapable of fusing with the oocyte plasma membrane

**DOI:** 10.17912/micropub.biology.000372

**Published:** 2021-02-21

**Authors:** Jun Takayama, Tatsuya Tajima, Shuichi Onami, Hitoshi Nishimura

**Affiliations:** 1 Laboratory for Developmental Dynamics, RIKEN Quantitative Biology Center, Kobe, Hyogo 650-0047, Japan; 2 Department of Frontier Studies of Medical AI, Tohoku University School of Medicine, Sendai, Miyagi 980-8575, Japan; 3 Department of Life Science, Faculty of Science and Engineering, Setsunan University, Neyagawa, Osaka 572-8508, Japan; 4 Life Science Center for Survival Dynamics, Tsukuba Advanced Research Alliance, University of Tsukuba, Tsukuba, Ibaraki 305-8577, Japan; 5 Laboratory for Developmental Dynamics, RIKEN Center for Biosystems Dynamics Research, Kobe, Hyogo 650-0047, Japan

## Abstract

*C. elegans spe-9* class genes encode sperm proteins with indispensable roles during fertilization. We have previously reported that *spe-45* belongs to the *spe-9* class, based on the finding that self-sperm of *spe-45(tm3715)* hermaphrodites were not consumed by fertilization. In this study, we directly observed live fertilization in the spermatheca of *fem-1(hc17)* females after mating with *spe-45(tm3715)* males. As expected, it was clearly shown that *spe-45* mutant spermatozoa failed to fuse with the oocyte plasma membrane. Thus, our live imaging system for *C. elegans* fertilization seems to be useful for evaluation of the functions of male and female gametes.

**Figure 1 spe-45(tm3715) spermatozoa fail to fuse with oocytes in the spermatheca of fem-1(hc17) females:**
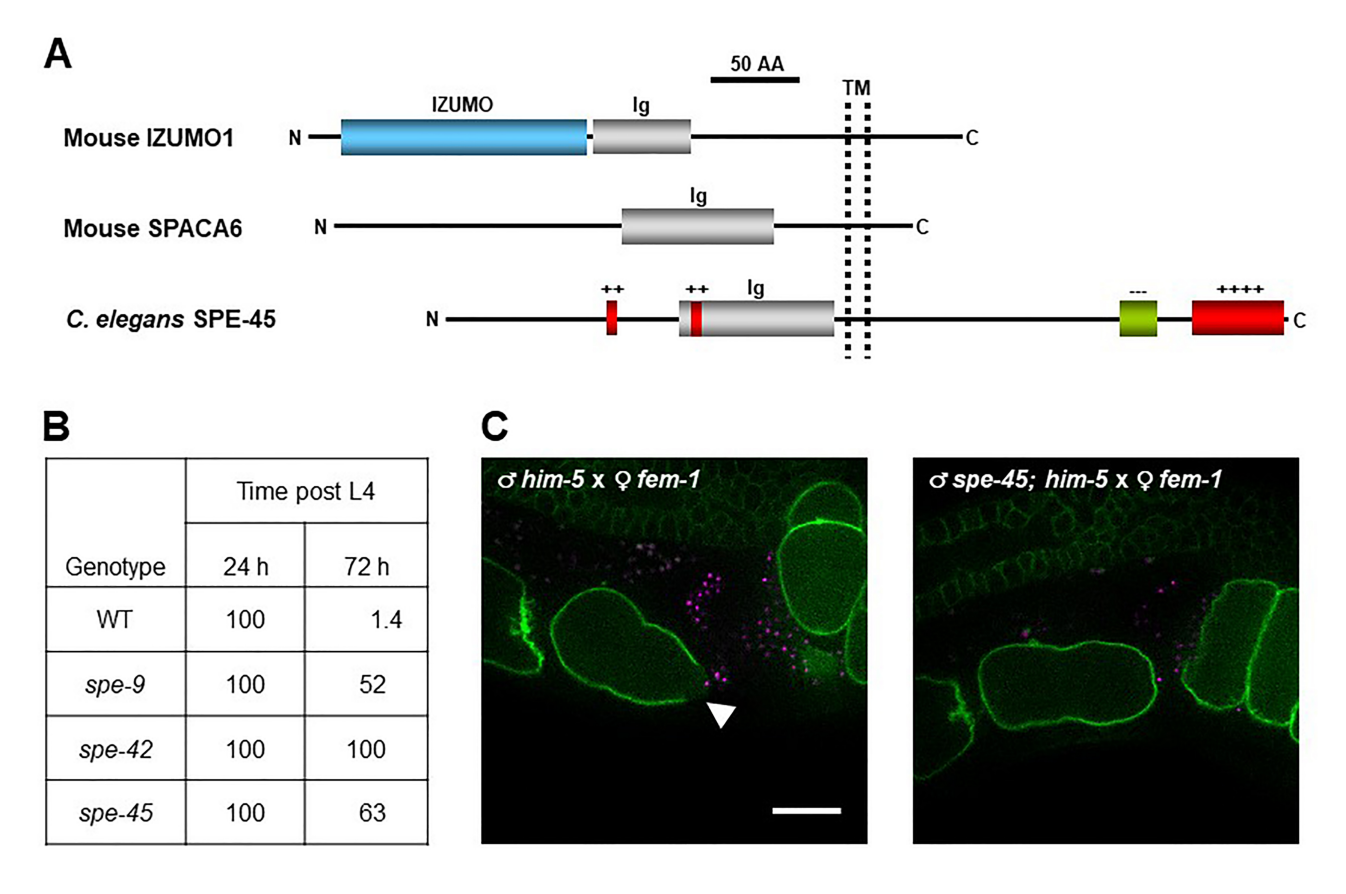
(**A**) Gross structures of mouse IZUMO1 and SPACA6 and *C. elegans* SPE-45. All the proteins shown here are sperm transmembrane (TM) proteins with a single immunoglobulin (Ig)-like domain that are essentially required for gamete fusion. The domain architectures of each protein were predicted by the SMART program (http://smart.embl-heidelberg.de). AA, amino acid; N, the amino-terminus; C, the carboxy-terminus; +, positively charged region; and –, negatively charged region. Numbers of “+” and “–” symbols represent relative numbers of basic and acidic amino acids, respectively. This panel was prepared based on the previous reports (Singaravelu *et al.*, 2015; Nishimura *et al.*, 2015; Nishimura and L’Hernault, 2016). (**B**) Time-lapse analysis on the number of self-sperm in the spermatheca of *spe-45* and other *spe-9* class mutant hermaphrodites. Data shown in this table are based on the assumption that the number of self-sperm in each spermatheca of N2 (wild type, WT), *spe-9(eb19)*, *spe-42(tm2421)*, and *spe-45(tm3715)* worms at 24 h post the fourth larval stage (L4) is 100%. This table was prepared according to the previously reported data (Nishimura *et al.*, 2015). (**C**) Live imaging of fertilization between WT or *spe-45* mutant spermatozoa and WT oocytes. We generated *oxIs318; him-5(e1490)* and *oxIs318; spe-45(tm3715); him-5(e1490)* males as WT and *spe-45* mutant, respectively, and these two strains produce spermatozoa of which nuclei are fluorescently labeled with mCherry (magenta). We also raised *fem-1(hc17); ltIs38* hermaphrodites at 25°C as WT females, in which the oocyte plasma membrane (PM) carries a green fluorescent protein (GFP, green). After mating of the females with WT (left) or *spe-45* mutant (right) males, live fertilization occurring in the spermatheca was observed by two-color 3D time-lapse spinning-disc confocal microscopy, as previously reported (Takayama and Onami, 2016). Still images were then acquired from movies recording the live fertilization. The white arrowhead indicates a fluorescence gap, which presumably shows that sperm-oocyte fusion occurs at the site. While the fluorescence gap region constantly appeared on the oocyte PM during fertilization by WT spermatozoa (n = 8), *spe-45* mutant spermatozoa could not yield the arc-shaped GFP signals on the oocyte surface (n = 8). Scale bar, 20 µm.

## Description

Gamete fusion is a pivotal step during fertilization to create an organism of the next generation. In *C. elegans*, since oocytes have no thick egg coat like the zona pellucida, perhaps spermatozoa directly bind to and fuse with the oocyte plasma membrane (PM). Thus, *C. elegans* is an excellent model to investigate how a spermatozoon and an oocyte fuse together.

*C. elegans spe* genes encode proteins needed for male germline functions, such as spermatogenesis (meiosis to produce spermatids), spermiogenesis (transformation of spermatids into spermatozoa), and fertilization (gamete fusion) (L’Hernaut, 1997; Nishimura and L’Hernault, 2010; Singaravelu and Singson, 2011). In particular, *spe-9* (Singson *et al.*, 1998), *spe-38* (Chatterjee *et al.*, 2005; Singaravelu *et al.*, 2012), *spe-41*/*trp-3* (Xu and Sternberg, 2003; Singaravelu *et al.*, 2012; Takayama and Onami, 2016), *spe-42* (Kroft *et al.*, 2005), and *spe-49* (Wilson *et al.*, 2018) belong to the *spe-9* class, and these genes all play essential roles during fertilization. However, it has been unclear whether members of the *spe-9* class have evolutionarily conserved roles during fertilization. To explore this question, we hypothesized that *C. elegans* has an ortholog of mouse *Izumo1*. This gene encodes a sperm transmembrane (TM) protein with a single immunoglobulin (Ig)-like domain that is required for sperm-oocyte fusion (Figure 1A) (Inoue *et al.*, 2005; Inoue *et al.*, 2015). Recently, mouse *Spaca6* was also identified as a testis-enriched gene encoding an Ig-like TM protein with an indispensable role in gamete fusion, as is the case for mouse *Izumo1* (Figure 1A) (Lorenzetti *et al.*, 2014; Barbaux *et al.*, 2020; Noda *et al.*, 2020). Moreover, Ig-like proteins from various species are known to act in gamete interactions (Nishimura and L’Hernault, 2016). Thus, we searched for male germline-enriched genes encoding proteins with the *Izumo1*-like domain architecture in the *C. elegans* genome by reverse genetic approaches (Nishimura *et al.*, 2015). *spe-45* (formerly named *F28D1.8*/*oig-7*) meets these criteria (Figure 1A), and *spe-45(tm3715)* hermaphrodites exhibit typical Spe phenotypes; the mutant worms are self-sterile but fertile after outcrossing to wild-type (WT) males. Singaravelu *et al.* also identified the same gene by a forward genetic approach (Singaravelu *et al.*, 2015).

Among the male germline functions, spermatogenesis and spermiogenesis are not impaired in *spe-45(tm3715)* worms (Singaravelu *et al.*, 2015; Nishimura *et al.*, 2015). As shown in Figure 1B, while self-sperm mostly disappeared in the spermatheca of WT hermaphrodites at 72 h post the fourth larval stage (L4), more than half of self-sperm still reside in the spermatheca of *spe-9(eb19)*, *spe-42(tm2421)*, and *spe-45(tm3715)* hermaphrodites even at the same time point (Nishimura *et al.*, 2015). Thus, all the tested mutants are presumably incapable of completing fertilization (gamete fusion), indicating that *spe-45* is a member of the *spe-9* class. Additionally, a domain-swapping approach showed that the Ig-like domains of *C. elegans* SPE-45 and mouse IZUMO1 share a conserved role during fertilization despite only ~9% identity of the entire amino acid sequences of these two proteins (Nishimura *et al.*, 2015).

The data in Figure 1B provide strong but indirect evidence that *spe-45* is involved during gamete fusion. On the other hand, we have succeeded in live imaging of fertilization occurring in the spermatheca by two-color 3D time-lapse spinning-disc confocal microscopy (Takayama and Onami, 2016). In this system, we used spermatozoa from males carrying the *oxIs318* allele, which produces mCherry-labeled sperm nuclei, and oocytes from *ltIs38* hermaphrodites, of which the PM is tagged with a green fluorescent protein (GFP). Therefore, we produced *him-5(e1490)* and *spe-45(tm3715); him-5(e1490)* males in the *oxIs318* background as WT-like and *spe-45* mutant, respectively, and *fem-1(hc17)* hermaphrodites in the *ltIs38* background as WT-like females.

After mating of *fem-1* females with WT or *spe-45* males, the spermatheca of the females was observed. From movies recording the live fertilization (see **Extended Data**), we could obtain still images showing a fluorescence gap on the oocyte PM, which was yielded by WT but not *spe-45(tm3715)* spermatozoa (Figure 1C). The place indicated by the fluorescence gap region seemed to represent where gamete fusion occurs, based on the previous report (Takayama and Onami, 2016). Therefore, our present findings show direct evidence that *spe-45(tm3715)* spermatozoa have a defect in sperm-oocyte fusion.

One of the advantages of using *C. elegans* for reproductive biology is that live fertilization in the spermatheca can be observed through transparent cuticles. Our imaging analysis demonstrates that spermatozoa lacking *spe-45* fail to fuse with the oocyte PM, unlike WT spermatozoa. However, it remains to be clarified how each *spe-9* class gene acts during gamete fusion. For instance, the data in Figure 1B imply that functional roles of *spe-9* and *spe-45* might be distinguishable from that of *spe-42* during gamete fusion. Our imaging system would provide important clues to solve the point.

## Methods

**Worm maintenance and culture**

We maintained and cultured worms at 20ºC unless otherwise stated, according to standard methods (Brenner, 1974). *him-5(e1490)* was used as WT to produce males. *spe-45(tm3715);* *him-5(e1490)* males were obtained by picking GFP-negative males from the SL1491 strain. To generate males from strains without the *him-5(e1490)* background, we incubated L4 hermaphrodites at 32ºC for 5 h, and the resulting F1 males were picked to use for further experiments. As *fem-1(hc17)* worms were used as WT females, fertilized eggs of the strain were cultured at 25ºC until they reached the adult stage.

**Microscopy**

Most worm handling, such as picking and dissecting, were carried out under SZ61 or SZX10 dissecting microscopes (Olympus). To observe GFP signals on the oocyte PM for a screening of the HTN3 strain, we used an Olympus BX53 fluorescent microscope. For imaging experiments, a Nikon Ti-E microscope with the objective lens Plan Apo VC 60xA/1.20 W (Nikon) was used. Confocal microscopy was performed with the spinning-disk confocal unit CSU-X1 (Yokogawa Electric Corp.). The confocal unit was equipped with a solid-state laser lines (488 nm and 561 nm; ALC-501 AOTF, Andor Technology). Time-lapse images were acquired with an EM-CCD camera (iXon DU-897, Andor Technology). Scanning along the z-axis in 3D time-lapse imaging was performed using a piezo stage-positioning system (Nano-Drive, Mad City Labs). Settings for time-lapse imaging were as follows: 18 planes of 0.5 µm z-axis interval, 2 s time interval and 40 ms exposure for each color. The camera and the piezo system were synchronized using a precision controller unit (PCU-100, Andor Technology). Imaging systems were controlled using iQ software (Andor Technology).

**Imaging of fertilization in the spermatheca**

To obtain females, we raised *fem-1(hc17); ltIs38* hermaphrodites at 25ºC. The feminized worms were mated with *oxIs318; him-5(e1490)* (WT) or *oxIs318; spe-45(tm3715); him-5(e1490)* (*spe-45* mutant) males at 25ºC overnight (female:male = 1:4). Then, the females after mating were immobilized using polystyrene nanoparticles and agarose pads as previously described (Takayama and Onami, 2016). Fertilization occurring in the spermatheca of the immobilized worms was observed by two-color 3D time-lapse spinning-disc confocal microscopy.

## Reagents

**Table:** 

**Strain**	**Genotype**	**Source**
DR466	*him-5(e1490) V*	CGC
BA17	*fem-1(hc17ts) IV*	CGC
SL1491	*spe-45(tm3715) IV; him-5(e1490) V; ebEx15-6[spe-45(+)(WRM066cF06) +* *myo-3p::gfp]*	Dr. L’Hernault
OD58	*unc-119(ed3) III; ltIs38[pie-1p::gfp::PH(PLC1delta1) +* *unc-119(+)]*	CGC
EG4883	*oxIs318[spe-11p::mCherry::H2B +* *unc-119(+)] II;* *unc-119(ed3) III*	CGC
HTN3*	*fem-1(hc17ts) IV; ltIs38[pie-1p::gfp::PH(PLC1delta1) + unc-119(+)]*	This study
HTN4*	*oxIs318*[*spe-11p::mCherry::H2B + *unc-119(+)]* II; him-5(e1490) V*	This study
HTN5*	*oxIs318*[*spe-11p::mCherry::H2B + *unc-119(+)]* II; spe-45(tm3715) IV; him-5(e1490) V;* ebEx15-6[spe-45(+)(WRM066cF06) + myo-3p::gfp]	This study
*HTN3, HTN4 and HTN5 might contain the *unc-119(ed3)* allele, but genotype of *unc-119* is not determined in these strains.		
**Primer**	**Nucleotide sequence**	
HN63	5’-ACTCGAACCAATTGGCAGGT-3’	
HN64	5’-CAAGGCGCACACTCTCTTTC-3’	
NM3887	5’-ACCGGAAACCAAAGGACGAGAG-3’	
NM3888	5’-ACGCCCAGGAGAACACGTTAG-3’	

To generate HTN3, we outcrossed BA17 hermaphrodites to OD58 males at 20ºC. F1 progeny were allowed to produce self-progeny. The F2 offspring were individually separated and allowed to produce F3 self-progeny. We individually picked five animals at the first larval stage (L1) among F3 progeny in each F2 plate, and the F3 larvae were cultured at 25ºC until they developed into adult worms. If all the five F3 worms from the same F2 plate became self-sterile, we judged that the F2 worm homozygously carries the *fem-1(hc17)* allele. In the F2 *fem-1(hc17)* worms, we also determined the genotype of *ltIs38* by observing GFP-signals on the oocyte PM under an Olympus BX53 fluorescent microscope. A difference of GFP-signal intensities can be distinguishable between heterozygote and homozygote of *ltIs38*.

To generate HTN4, we outcrossed EG4883 hermaphrodites to DR466 males. F1 progeny were allowed to produce self-progeny. The F2 offspring were individually separated and allowed to produce F3 self-progeny. If a certain F2 plate contains sons as F3 worms, the genotype of *oxIs318* in the F2 worm was determined by single-worm PCR (Nishimura **et al.*,* 2015), using a primer set of NM3887 and NM3889 (Frøkjær-Jensen *et al.*, 2008).

To generate HTN5, we outcrossed EG4883 hermaphrodites to SL1491 males. GFP-positive F1 progeny were picked and then allowed to produce self-progeny. GFP-positive F2 offspring were individually separated and allowed to produce F3 self-progeny. If a certain F2 plate contains sons as F3 worms, five GFP-negative F3 males were picked from the plate to examine the genotypes of *spe-45* and *oxIs318* by single-worm PCR, using primer sets of HN63 and HN64 for *spe-45* and NM3887 and NM3888 for *oxIs318*. If all the five tested F3 worms carried both *spe-45(tm3715)* and *oxIs318* as homozygotes, GFP-positive hermaphrodites in the same F2 plate were maintained as *oxIs318; spe-45(tm3715); him-5(e1490); ebEx15-6*. As *oxIs318; spe-45(tm3715); him-5(e1490)* worms were required, GFP-negative animals were picked.

**Extended Data:**

him-5 x fem-1.mov

Description: This is a movie file recording live fertilization between WT spermatozoa and WT oocytes.

spe-45; him-5 x fem-1.mov

Description: This is a movie file recording live fertilization between *spe-45* mutant spermatozoa and WT oocytes.

Extended Data are archived at CaltechData.
